# Supplementary material for: HIV-1 Gag gene mutations, treatment response and drug resistance to protease inhibitors: A systematic review and meta-analysis protocol
Source: PLoS One. 2021 Jul 1;16(7):e0253587. doi: 10.1371/journal.pone.0253587 (PMC8248685; doi:10.1371/journal.pone.0253587)
Supplement: S1 File — (DOCX) [file pone.0253587.s002.DOCX]

S1 File. Search Strategies

| Database | Query |
| --- | --- |
| PubMed | (((("HIV Infections" [MeSH Terms] OR ("HIV" [All fields] OR ("HIV-1"[All fields] OR ("HIV1" [All fields] OR HIV infect[All fields] OR ("Human immunodeficiency virus" [MeSH Terms] OR ("Human immunedeficiency virus" [All fields] OR ("human immuno-deficiency virus [All fields] OR ("human immune-deficiency virus" [All fields] OR ("human immun" AND "deficiency" virus[All fields]) OR "acquired immunodeficiency syndrome" [MeSH Terms] OR ("acquired immunedeficiency syndrome" [All fields] OR("acquired immuno-deficiency syndrome" [All Fields] OR ("acquired immune-deficiency syndrome" [All Fields] OR ("acquired immun") AND "deficiency syndrome" [All Fields] OR" Antiretroviral Therapy"[MeSH Terms] OR ("Highly Active" [All Fields] OR ("Anti-Retroviral Agents" [All Fields] OR ("Antiviral Agents" [All Fields] OR ("anti" AND (HIV[All Fields])) OR ("antiretroviral[All Fields] OR ("anti" AND "retroviral" [All Fields]) OR ("HAART" [All Fields] OR ("ART[All Fields] OR ("anti" AND "acquired immunodeficiency" [All Fields]) OR ("anti" AND "acquired immunedeficiency " [All Fields]) OR ("anti" AND "acquired immuno-deficiency" [All Fields]) OR ("anti" AND "acquired immune-deficiency" [All Fields]) OR ("anti AND "acquired immune") AND (deficiency[All Fields]))))  AND  (Antiretroviral [All Fields] OR ("antiretroviral therapy, highly active"[MeSH Terms] OR ("antiretroviral"[All Fields] AND "therapy"[All Fields] AND "highly"[All Fields] AND "active"[All Fields]) OR "highly active antiretroviral therapy"[All Fields] OR "haart"[All Fields]) OR ("antiretroviral therapy, highly active"[MeSH Terms] OR ("antiretroviral"[All Fields] AND"therapy"[All Fields] AND "highly"[All Fields] AND "active"[All Fields]) OR "highly active  antiretroviral therapy"[All Fields] OR ("highly"[All Fields] AND "active"[All Fields] AND"antiretroviral"[All Fields] AND "therapy"[All Fields])) OR (antiretrovirus[All Fields] AND agent[All Fields]) OR (anti[All Fields] AND ("retroviridae"[MeSH Terms] OR "retroviridae"[All Fields] OR"retrovirus"[All Fields]) AND agent[All Fields])))  AND  ((("Group-specific antigen "[MeSH Terms] OR ("Gag"[All Fields] OR ("Group specific antigens" [All Fields] OR "Gag cleavages sites"[All Fields] OR ("Gag-pol"[All Fields] AND ("Protease inhibitors"[All Fields] OR ("anti-Protease" OR ("Protease inhibitors boosted by ritonavir"[MeSH Terms] OR ("PI/r"[All Fields])))  AND  ((("HIV drug resistance" [MeSH Terms] OR ("drug resistance mutations" [All Fields] OR ("mutation [All Fields] OR ("resistance" [All Fields] OR "resistance associated mutations" [MeSH Terms] AND ("HIV-Protease" [All Fields])) OR "Protease [All Fields] OR "Protease strand transfer inhibitors"[MeSH] OR" Lopinavir" [All Fields] OR "Atazanavir" [All Fields] OR " Darunavir "[All Fields"])))  AND  ("2000/01/01"[PDAT]: "2020"[PDAT]) |
| Embase  (Ovid interface) | 1.exp Human immunodeficiency virus/  2.exp Human immunodeficiency virus infection/  3.(hiv or hiv?1 or human immun#deficiency virus or human immun#-deficiency virus or (human immun# adj3 deficiency virus) or acquired immun#deficiency syndrome or acquired immun#-deficiency syndrome or (acquired immun# adj3 deficiency syndrome)).tw.  4. or/1-3  5. exp Group-specific antigen/  6. Group specific antigen/  7. exp Gag/  8.exp Gag cleavages sites/  9. or/5-8  10. exp treatment failure/  11. exp treatment failed/  12.exp Therapeutic failure/  13.exp treatment response/  14. or/10-12  15. exp Protease inhibitors/  16. exp Protease inhibitors boosted by ritonavir/  17. exp PI/  18. exp PI/r/  19. or/15-18  20. exp HIV drug resistance/  21. exp drug resistance mutations/  22.exp resistance/  23. exp resistance associated mutations/  24. or/20-23  25. 4 and 9  26. 9 and 24  27 limit 26 to yr="2000 - 2020" |
| CINAHL | S1 (MH "Antiretroviral Therapy, Highly Active")  S2 (MH "Anti-Retroviral Agents+")  S3 (MH "Antiviral Agents")  S4 TI ( (anti and HIV) OR antiretroviral* OR (anti and retroviral*) OR HAART OR cART OR (anti and acquired immunodeficiency) OR (anti and acquired immunedeficiency) OR (anti and acquired immuno-deficiency) OR (anti and acquired immun* and deficiency) ) OR AB ( (anti and hiv) OR antiretroviral* OR (anti and retroviral*) OR HAART OR cART OR (anti and acquired immunodeficiency) OR (anti and acquired immunedeficiency) OR (anti and acquired immuno-deficiency) OR (anti and acquired immun* and deficiency)  S5 S1 OR S2 OR S3 OR S4  S6 (MH "HIV Infections+")  S7 (MH "Human Immunodeficiency Virus+")  S8 TI (HIV OR HIV 1 OR human immunodeficiency virus OR human immuno-deficiency virus OR acquired immunodeficiency syndrome OR acquired immunodeficiency syndrome OR (acquired immuno-deficiency syndrome) ) OR AB ( HIV OR HIV 1 OR human immunodeficiency virus OR human immuno-deficiency virus OR acquired immunodeficiency syndrome OR acquired immuno-deficiency syndrome or (acquired immuno deficiency syndrome)  S9 (MH "Sexually Transmitted Diseases, Viral+")  S10 S6 OR S7 OR S8 OR S9  S11 TI ("HIV drug resistance" OR "drug resistance mutations" OR mutation OR resistance OR "resistance associated mutations")  S12 TI ("HIV-Protease" OR Protease OR "Protease inhibitors" OR Lopinavir OR Atazanavir OR Darunavir OR "Treatment response")  S13 S5 OR S10  S14 S11 AND S12 AND S13  S15 (MH "Africa+")  Limiters - Published Date: 2000/01/01-2020 |
