# Supplementary material for: HIV-1 Gag gene mutations, treatment response and drug resistance to protease inhibitors: A systematic review and meta-analysis protocol
Source: PLoS One. 2021 Jul 1;16(7):e0253587. doi: 10.1371/journal.pone.0253587 (PMC8248685; doi:10.1371/journal.pone.0253587)
Supplement: S2 File — (DOCX) [file pone.0253587.s003.DOCX]

**S2 File**. Assessing the quality of evidences and the strength of recommendations.

| **Types of studies** | **Risks of bias** | **Interpretation** | **Quality of evidence** | **Strength of the recommendation** |
| --- | --- | --- | --- | --- |
| Randomized studies | Low risk of bias | Most information is from studies at low risk of bias. | High | Strong |
|  | Unclear risk of bias | Most information is from studies at low or unclear risk of bias. | Moderate | Moderate |
|  |  |  | Low |  |
| Non-randomized studies | High risk of bias | The proportion of information from studies at high risk of bias is sufficient to affect the interpretation of results. | Very Low | Weak |
